# The Care of the Patient during the Lying-in Period

**Published:** 1892-09

**Authors:** Margaret Caldwell

**Affiliations:** Waukeshee, Wis.


### THE CARE OF THE PATIENT DURING THE LYING-IN PERIOD.*

BY MARGARET CALDWELL, M. D., WAUKESHEE, WIS.

That branch of the subject of obstetrics which I have chosen for my paper, "The Care of the Patient During the Lying-in Period," may seem too trivial with which to take up your time, especially as many physicians make no call after confinement, and a large number make but one. Both of these classes say, "Send for me if I am needed;" only the minority see the patient through the period of convalescence, which, in ordinary cases, is marked by the full complement of milk, a good appetite, the natural condition of the excretory organs, and later on, the proper position, etc., of the uterus. I am stating the subject as it has come under my own observation. If a labor is so free from complications that no call, or but one, is necessary after confinement, I think that patient would most likely be safer without a doctor ; then there is no chance of carrying germs into the vagina during examination. The danger of infection in the two first stages of labor is great, when the temptation exists to pass the examining finger up between the head and the tender cervix, perhaps to confirm a former diagnosis of position, or without a purpose ; the temptation is there, and very few, who are not experts, but will yield to it.

When the physician is called, if the patient's bowels have not moved since the commencement of labor, an enema should be given at once; then a bath—either a full, sitz, foot or sponge bath. If the necessary cleanliness of the body has been attended to, a foot bath will do more than drugs to relieve nervousness ; it removes the nervous, nagging, irregular pains, which wear the patient out, and it facilitates labor in many ways. After the bath clothe the limbs in warm stockings and a comfortable undergarment, woolen preferred ; there will not be so much danger from taking cold at the close of

*Read before the Wisconsin State Medical Society.

labor from the necessary exposure, and with one of Howard Kelley's abdominal pads, no stain is left upon bed or upper clothing, no changing afterward becoming necessary—only the removal from the lower limbs.

It seems to me that one great source of infection during labor is from allowing some of the enclosed air under the bed clothing to enter the vagina, possibly the uterus. Those cases are rare, but I have had a few where the vagina stood broad open for a certain period of time after the expulsion of the child. It is wise to anticipate this, by closing the vulva behind the child as it emerges from the parturient canal.

If, after proper antiseptic and aseptic procedure, with the uterus firm and of good size when grasped by the external hand, and an examination of the secundines shows that the membranes have been wholly expelled with the placenta intact, there is no need of furthei exploration, and the accoucheur need have no fear of sepsis from the uterus. This sounds like truth, but it is not. I had one case that deceived me. She developed high temperature on the third day, with all signs and symptoms of septic infection. An intra-uterine exploration revealed numerous elevations on the right posterior side, which could not be removed with the dull curette without danger of going through the uterine wall. Gentle scraping removed what seemed to be placental tissue. The scraping, followed by an antiseptic intra-uterine douche, would bring the temperature down to normal. I found it necessary to go through that routine, at first every day, then every other day for two weeks, when the uterine wall became smooth, and the septic symptoms did not return. This was her fourth confinement, in all of which I had attended her, and had no trouble before. She said she had had an abortion of two months previous to her last conception, for which she had had no doctor.

Was the debris the result of the abortion? She had her fifth confinement a few months since, which could not be improved upon. If the labor has been tedious, I give an aseptic vaginal douche; if complicated, making many examinations necessary, or the use of instruments, etc., I give an antiseptic douche. I am usually guided by the patient's feelings about the continued use of the douche; always use it if the lochia is disagreeable, and once or twice daily, if it is soothing. It is wise to ascertain if the nurse in charge understands the meaning of the term.

Let me illustrate. A practitioner in Chicago told me that after giving to the husband, who was acting as nurse, the usual order, "Let me know if everything is not all right," left. In the course of a few days, the nurse called on the doctor to ask if the foul odor could not be stopped. He said, "Did you use the syringe I gave you?" "Oh! yes, doctor, and although it does not affect the odor, it moves the bowels finely." Another case, which was under the care of a brother practitioner in the town in which I live. The doctor took a Davidson syringe, showed the intelligent (?) nurse how to use it in a basin of water, and left happy. Next day, not liking the appearance of the patient, he inquired, "Did you use the syringe?" "Yes, doctor, but the mixture you ordered was no sooner down, than it all came up again, and everything that had been eaten for a week." It is a fact that the nurse forced a pint of soap-suds down the patient's throat, by means of the syringe.

Repair all vulvar lacerations. It prevents many of the so-called uterine diseases, and the slightest tear where the cuticle and mucous membranes meet, may give rise to sepsis, by absorption of the lochia, and for a day or two, will be very distressing when the patient urinates. If any stitches are necessary, I have the douche given, when urination takes place. It dispenses with the use of the catheter, which is always a source of discomfort at best. A good sized piece of aseptic cotton, placed over the vulva, need be changed only when the patient urinates. Napkins placed over the cotton, may be changed as often as necessary for cleanliness, according to the amount of the flow. In ordinary cases, I allow the patient to decide about the abdominal bandage. I prefer it. I think it lessens the tendency to hemorrhage, cuts short after pains, and is a support to the patient. A napkin of four or five thicknesses, saturated with hot arnica or alcohol, placed under the bandage, is agreeable, and a good help in relieving after-pains; a sponge bath, with a little alcohol in the water, given in the evening, is refreshing and conducive to sleep. The physician should not leave the house for at least an hour, if possible, after the completion of labor, or until the uterus is firmly contracted, and no danger of hemorrhage apparent. Let the patient have all the cold water to drink she wants, and give light foods till the mammary secretion is established.

The food during lactation, need be restricted only to those foods which are thoroughly digested by the mother; if they agree with the mother they will agree with the child. A bowl of gruel set by the bedside, and drank by the mother, when the child requires the breast during the night, will prevent that thirst and weariness which all nursing mothers complain of in the morning. A gentle purge should be given the second or third day; castor oil, I prefer. If its name should be changed, no patient would object to it, and no patient could detect its taste in a properly constructed mixture, the refining process has been brought so near perfection; but we all know it is more easily prescribed than accepted. The patient should not be dismissed until the physician has made a thorough examination, after sufficient time has elapsed for the generative organs to assume their natural condition, which is never less than six or eight weeks.

How many patients, when presenting themselves to a physician, say that they have not been well since a certain confinement, owing to cervical lacerations, sub-involution or some one of the many results which may arise after the most normal labor, and in the hands of a genuine expert. Each one of you, with a moment's thought, can call to mind a number of homes where the mother, who should be the life and sunshine of the domestic circle, is a constant sufferer, because some doctor did not see that she was all right, after passing through that fiery ordeal, which crowned her with the glorious title of motherhood, than which there is none greater this side of heaven.

